# Some Scientific Aspects of Insanity

**Published:** 1892-01-09

**Authors:** 


### Some Scientific Aspects of Insanity.

### VIII.—The Care and Treatment of Melancholia.

Only those who have suffered from mental depression of more than ordinary severity are able to form any adequate idea of the misery and suffering which is endured by melancholic patients. Most civilized people who are gifted with a tolerably good imagination are able to sympathise with bodily pain, but I fear that it is only within the power of a few very highly gifted individuals to imagine, and, therefore, fully to sympathise with the terrible sufferings of the mentally depressed. The suffering of the body is but insignificant compared with that of the mind, which has an unlimited capacity for intensity and persistence of painful feelings. Those of us who are incapable of fully realising these depths of misery should attempt to extend a sympathy based upon the external symptoms of melancholia.

This painful state is due, in great measure, to a lack of nervous tone, and in an equal measure does the body suffer with the mind. In the treatment of melancholia there are two great aims to be kept in view. There is first of all the alleviation of the mental pain, and secondly, the restoration of the bodily health.

With regard to the alleviation of mental pain, it is, of course, difficult to lay down any rules, except of the most general kind. It ought to be the aim of every attendant and nurse to distract the patient's thoughts from constantly brooding upon his own morbid condition. This is best achieved by endeavouring to get the patient to engage in some light, simple piece of work, however mechanical or trifling it may be, and by persevering endeavours, from day to day, gradually to increase the extent and the character of the work. The amusement of the patient is only secondary as a curative agent to his employment. The patient's surroundings ought to be made as cheerful as possible. The furniture of the ward, and the knick-knacks and ornaments ought to be as bright as possible, and as advantageously arranged as circumstances will permit. A melancholic patient will make no effort to amuse himself. The effort must, therefore, be made for him by others, and he should be induced against his inclination to participate in any amusement which, consistently with special medical orders, can be provided for him by his attendant. A melancholic patient should never be forced to engage in work, or in amusement which is manifestly distasteful to him.

The manner of the attendant in dealing with the patient is a matter of no little importance. It might be urged that the cup of misery of these patients is so full that an unkind word or a discourteous attitude on the part of an attendant is of little consequence. It is true that a melancholic patient desires above all things, to be alone with his misery, and pays very little regard to the manners of others. Yet when the patient is brought to a curative hospital, and when the good work is begun, every kind action, every courteous word spoken by an official or an attendant must have a powerful influence for good even upon those self-absorbed, selfish, suffering beings.

It is very useless to lose one's temper with a melancholic patient; it is worse than useless, it is brutal to treat one unkindly. It is foolish to argue with them, and to tell them there is nothing the matter, for their health is seriously disordered, and no amount of argument can put it right.

Secondly, the restoration of bodily health. Like many other diseases, such as fevers, as much or more can be done by the skilful nursing of the patient than by the medical treatment. The medical officers may prescribe food and medicine, but only the attendant can see to these being properly administered. Above all, it is requisite in the treatment of melancholia strictly to observe that the patient eats a sufficient quantity of good, nourishing food. Not only has the attendant to observe and report that this has been done, but he must observe and report it accurately. It is not sufficient to report in a general way that the patient has taken a meal. It is an absolute duty to be able to state the exact quantity of each article of diet which the patient has consumed. It must be borne in mind that melancholic patients will use every endeavour to avoid taking a sufficient quantity of food. When the patient absolutely refuses food the attendant must endeavour first by persuasion to induce him to do so, and if that fails a gentle attempt must be made to feed him with the spoon. No force is to be used on any pretext whatever. If the patient still refuses to be fed, the matter should at once be reported to the medical officer in charge.

The care of melancholic patients is one of the most important duties which falls to the lot of asylum officials. A person incapacitated by mental disease from taking care of his own life is placed in an asylum with the object of guarding against suicide. If, through the careless neglect of those immediately in charge the patient succeeds in putting an end to his life, the responsibility of his death lies directly at the door of his guardians. It seems to me that the patient's death is in these circumstances as distinctly attributable to his personal attendant, as if that attendant had directly murdered him. Of equal importance, therefore, with the measures taken to cure the patient are the measures which should be taken to protect his life. There is, of course, only one obvious way of insuring immunity from this dreadful calamity of suicide, and that is never to lose sight of the patient for a moment. A great many suicides in asylums have occured by mistakes in the transference of suicidal patients from the charge of one attendant to that of another. The duty of an attendant towards a melancholic patient does not cease until he has, without any chance of mistake, openly and formally handed over the patient to the care of the attendant who relieves him. There is a variety of melancholia which is called suicidal melancholia, and which I did not mention because I wish you distinctly to remember that every case of melancholia is suicidal.

